# Industry and geographic patterns of use and emission of carcinogens in Ontario, Canada, 2011–2015

**DOI:** 10.17269/s41997-018-0075-0

**Published:** 2018-05-29

**Authors:** Catherine E. Slavik, Sheila Kalenge, Paul A. Demers

**Affiliations:** 1 Occupational Cancer Research Centre, Cancer Care Ontario, Toronto, Ontario Canada; 2 School of Geography and Earth Sciences, General Sciences Building, McMaster University, 1280 Main Street West, Hamilton, Ontario L8S 4K1 Canada; 3 Dalla Lana School of Public Health, University of Toronto, Toronto, Ontario Canada

**Keywords:** Environmental carcinogens, Manufacturing and industrial facilities, Industry, Environmental exposure, Prevention and control, Cancérogènes environnementaux, Installations industrielles et de fabrication, Industrie, Exposition environnementale, Prévention et contrôle

## Abstract

**Objectives:**

The goal of this study was to leverage data from two environmental regulatory initiatives, Ontario’s Toxics Reduction Act (TRA) and Canada’s National Pollutant Release Inventory (NPRI), to assess their ability to monitor trends in the use and emission of carcinogens by industry sector in Ontario.

**Methods:**

Data reported to the TRA and NPRI by industrial facilities in Ontario were retrieved from 2011 to 2015. Twenty-six known and suspected carcinogens were identified (IARC) and the trends in the use and emission were evaluated by industry sector. The locations of industrial facilities that used and released carcinogens were mapped by Public Health Unit (PHU).

**Results:**

Chemical manufacturing and primary metal manufacturing sectors accounted for 84% of all reported industrial use of carcinogens during the period 2011–2015. The largest source of carcinogen emissions came from facilities in the primary metal manufacturing and paper manufacturing sectors. The largest number of industrial facilities that reported the use and release of carcinogens were located in the City of Toronto and Peel Region PHUs. Overall, the use of carcinogens across all sectors appeared to decrease by 8%, while emissions increased by about 2%.

**Conclusion:**

The results of this study show the need to reduce the use and emission of select carcinogens in priority industry sectors. Environmental reporting programs, such as the TRA and NPRI, can serve as important tools in cancer prevention by tracking potential carcinogen exposures in the environment and in the workplace.

## Résumé

**Objectifs:**

Exploiter les données de deux initiatives réglementaires environnementales, la *Loi sur la réduction des toxiques* (LRT) de l’Ontario et l’Inventaire national des rejets de polluants (INRP) du Canada, pour estimer leur capacité de surveiller les tendances d’utilisation et d’émission de cancérogènes par secteur d’activité en Ontario.

**Méthode:**

Nous avons récupéré les données déclarées à la LRT et à l’INRP par les installations industrielles de l’Ontario entre 2011 et 2015. Vingt-six cancérogènes connus et soupçonnés (selon le Centre international de recherche sur le cancer) ont été recensés, et les tendances d’utilisation et d’émission de ces substances ont été évaluées par secteur d’activité. Les emplacements des installations industrielles ayant utilisé et rejeté des cancérogènes ont été cartographiés pour chaque bureau de santé publique (BSP).

**Résultats:**

Le secteur de la fabrication de produits chimiques et celui des métaux de première fusion ont représenté 84% des utilisations industrielles déclarées de cancérogènes pour la période de 2011 à 2015. Les installations du secteur des métaux de première fusion et les papetières ont constitué la plus grande source d’émissions de cancérogènes. Nous avons recensé le plus grand nombre d’installations industrielles disant avoir utilisé et rejeté des cancérogènes dans les BSP de la Ville de Toronto et de la Région de Peel. Dans l’ensemble, l’utilisation de cancérogènes dans tous les secteurs d’activité semble avoir diminué de 8%, mais les émissions ont augmenté d’environ 2%.

**Conclusion:**

Les résultats de l’étude montrent qu’il faut réduire l’utilisation et les émissions de cancérogènes ciblés dans des secteurs d’activité prioritaires. Les programmes de rapports sur l’environnement comme la LRT et l’INRP peuvent être d’importants outils dans la prévention du cancer en localisant les expositions possibles aux cancérogènes dans l’environnement et sur les lieux de travail.

## Introduction

About one out of every two Canadians will be diagnosed with cancer in their lifetime (Canadian Cancer Society’s Advisory Committee on Cancer Statistics 2017). However, according to the Canadian Cancer Society, approximately half of all cancer deaths can be prevented (Canadian Cancer Society 2014). In Ontario, it is estimated that over 3000 cancer cases could be prevented each year if exposures to carcinogens in the workplace were reduced (Cancer Care Ontario, Occupational Cancer Research Centre 2017), and an additional 1970 cases if exposures to industrial carcinogens were reduced (Cancer Care Ontario, Ontario Agency for Health Protection and Promotion 2016). These estimates demonstrate the need to reduce the industrial use and emission of carcinogens in Ontario. One approach to achieving reductions constitutes tracking toxic use and emissions (Jacobs et al. 2014).

Chemical releases have been systematically monitored across various industrial facilities for some time in Canada, with environmental emissions data going as far back as 1993 (Environment and Climate Change Canada 2017). Facilities report their emissions of contaminants that may impact human health and the environment to Environment and Climate Change Canada’s (ECCC) National Pollutant Release Inventory (NPRI) (Environment and Climate Change Canada 2016). In Ontario, the Toxics Reduction Act of 2009 (TRA) requires industrial facilities in four major manufacturing and mineral processing sectors to track and report their use of toxic substances (i.e., amount entering the facility, amount created and amount contained in product) to the Ontario Ministry of Environment and Climate Change (MOECC) (Government of Ontario 2009). Ontario’s TRA was modeled after Massachusetts’s Toxics Use Reduction Act of 1989, which has led to significant reductions in the use and release of carcinogens by industrial facilities in that state by 32% and 93%, respectively, from 1990 to 2010 (Jacobs et al. 2014). Previously, we utilized data from Ontario’s TRA to examine carcinogen use and release trends by cancer site (Slavik et al. 2017).

Data from these environmental reporting programs can aid in assessing exposures in the workplace and in the environment by serving as surrogates for potential exposures. Reported contaminant emissions can serve as an indicator of an environmental hazard, while use can indicate a potential occupational hazard. In most cases, these environmental programs offer the only source of complete data that is publicly and freely available and remain a valuable source of information to support policy-making (Pulles 2008). Chemical exposures in the workplace have been identified as a priority research issue in Ontario that could help to inform occupational cancer prevention policies in the province (Hohenadel et al. 2011).

Previous studies using emissions data from environmental reporting programs have mostly used emissions data to explore issues of environmental justice relating to the location of facilities in communities with large populations of minorities (Neumann et al. 1998; Mennis and Jordan 2005), higher poverty levels (Ringquist 1997), and lower socio-economic status (Mennis 2002). Other research has used emissions data to examine potential health risks associated with exposure to industrial emissions, such as cancer and respiratory conditions (Chakraboty 2004; Wong et al. 2016). NPRI data have also been used to analyze emission trends and the distribution of industrial facilities in neighbourhoods by various socio-economic characteristics in Montreal and in Toronto (Premji et al. 2007; Kershaw et al. 2013).

A recent study by Setton et al. analyzed NPRI data for each province and found that approximately half of the reported toxic releases to air came from industrial sources in Ontario, making industries a significant contributor of air contaminants in the province compared to other sources such as road emissions (Setton et al. 2015). The only study that assessed trends in NPRI chemical releases using an industry sector approach was carried out two decades ago by Olewiler and Dawson, who found that nationwide, the four sectors that released the most toxic substances by weight were the chemical, primary metal, and petroleum and coal manufacturing sectors, and the mining sector (Olewiler and Dawson 1998). In Massachusetts, data from their toxic use reduction program was analyzed and facilities in the chemical manufacturing sector were found to be responsible for more than half the total use of all chemicals in the state, while no other sectors made up more than 10% of total use (Commonwealth of Massachusetts 2014). No study assessing sector trends in the use and release of industrial contaminants has ever been undertaken in Ontario and very little is known about the presence of specific carcinogens in particular industry sectors. The results of this study will fill a key knowledge gap in our understanding of worker exposures in specific industry sectors in Ontario and help to identify potential sectors where future cancer prevention efforts ought to be directed.

Since exposures to occupational carcinogens vary by industrial sector (Driscoll et al. 2004), the risk of developing cancer associated with specific exposures is often linked to particular sectors (Mannetje and Kromhout 2003). Analyzing data using industry sector classifications like the North American Industry Classification System (NAICS) can therefore serve as a useful tool for assessing potential occupational exposures to carcinogens and for directing cancer prevention efforts to priority sectors. Data from the TRA on the use of carcinogens in various industrial facilities can make this type of assessment of exposures by industry sector possible. The goal of this study was to leverage data from two environmental regulatory initiatives, Ontario’s TRA and Canada’s NPRI, to assess their ability to monitor trends in the use and emission of carcinogens by industry sector in Ontario. In addition, we use the data to examine the geographic location of industrial facilities reporting carcinogen use and release in the province.

## Methods

### Databases

Data on the use of toxic substances from industrial facilities reporting to the Ontario TRA were retrieved online from the MOECC’s website for the years 2011 to 2015 (Government of Ontario 2017). Data reported for the year 2010 (the first year of data collection after the program’s implementation) were omitted as the reporting requirements were different compared to subsequent years. The following variables were used: substance name and Chemical Abstract Service (CAS) number, facility NAICS code, facility postal code, number of employees, and the amount of toxic substances entering the facility referred to as “estimated use” (in tonnes [t]). Facilities reported “estimated use” ranges (i.e., > 0 to 1 t, > 1 to 10 t, etc...), and the mid-point of the range was used for statistical purposes.

Emissions data from industrial facilities reporting to Canada’s NPRI were also retrieved online from ECCC’s website as a bulk dataset for data from all facilities reporting since 1993 (Environment and Climate Change Canada 2017). Data was limited to Ontario (2011–2015) and the following variables were used: year, substance name and CAS number, facility NAICS industry code (31-33, 212), facility postal code, number of employees, and the amount of toxic substances released to air that was calculated as the sum of releases to air across multiple variable categories (i.e., stack or point releases, fugitive releases, spills, storage and handling, and other releases, in tonnes). We restricted our analysis to carcinogens released in air as releases to air have been identified as likely having a larger impact on human health, and inhalation is the primary route of exposure within the workplace, which is also a focus of this study (Lim et al. 2010). In addition, the data for air emissions are more consistently reported by industrial facilities, resulting in a more complete dataset.

### Data analysis

Industrial facilities in Ontario that report their releases to the NPRI must report their use of toxic substances to the MOECC’s TRA program from a list of substances prescribed by the program. Of the 322 substances prescribed under the TRA, those classified as known human carcinogens (group 1) and probable human carcinogens (group 2A) by the International Agency for Research on Cancer (IARC) were selected for analysis in this study (*n* = 26) (International Agency for Research on Cancer 2017). The extracted data from the 2011–2015 NPRI and TRA datasets were restricted to the 26 identified known and suspected carcinogens according to their CAS numbers. Although the NPRI program requires facilities to report on releases of the particulate matter less than 2.5 μm in diameter (PM_2.5_), particulate matter less than 10 μm (PM_10_), and total particulate matter, only releases of PM_10_ were selected for analysis in this study as they were reported more frequently. IARC has classified all particulate matter as a known human carcinogen (The International Agency for Research on Cancer (IARC) 2016).

A postal code conversion file program was used to convert the postal code of each industrial facility reporting to the TRA and to the NPRI into its respective Public Health Unit (PHU), in order to geographically analyze the data for industrial facilities in both datasets. There are 36 PHUs in all of Ontario (Ontario Ministry of Health and Long-Term Care 2014). The number of industrial facilities that reported the use and the release of carcinogens in 2015, the most recent dataset available at the time of this study, was identified for each PHU and mapped using ArcGIS 10.5.1 (Environmental Systems Research Institute 2017). Data was classified using the “natural breaks (jenks)” method to maximize the difference in numbers of facilities between PHUs on the maps.

The top 10 industry sectors were ranked by the amount of known and suspected carcinogens used and released which were summed for all years from 2011 to 2015. The top three substances used and released, as well as the mean number of employees, were also identified for each of the top 10 industry sectors. Additionally, the amount used and released in each sector, and the number of facilities in each sector, was identified for each year from 2011 to 2015. Carcinogen use was plotted by year for each sector and trends were calculated by dividing the slope of the trend line generated (for each sector’s use by year) by its intercept and multiplying by 100%. The same method was used to calculate trends for releases. All data analyses were performed using the statistical software SAS 9.4 (SAS Institute Inc. 2013, Cary, NC).

## Results

The total estimated use and release of carcinogens by industrial sector summed for all years from 2011 to 2015, the mean number of employees for each sector and the top three known and suspected carcinogens by amount used and released are shown in Table 1.

**Table 1:** Total estimated use and release of carcinogens and top carcinogens used and released by industrial sector, ranked by use and releases, TRA program 2011–2015, NPRI 2011–2015

Carcinogen use				Carcinogen releases		
Sectors using carcinogens	Mean employees	Total estimated use	Top carcinogens used	Sectors releasing carcinogens	Total releases	Top carcinogens released
Chemical manufacturing	12,819	10,468,540	Benzene; vinyl chloride; 1,3-butadiene	Primary metal manufacturing	23,165.75	Particulate matter; benzene; nickel
Primary metal manufacturing	132,401	4,749,630	Nickel; benzene; lead	Paper manufacturing	12,129.99	Particulate matter; formaldehyde; benzene
Petroleum and coal product manufacturing	14,891	1,977,480	Benzene; 1,3-butadiene; nickel	Mining (except oil and gas)	9874.80	Particulate matter; nickel; lead
Mining (except oil and gas)	28,461	658,310	Nickel; lead; arsenic	Non-metallic mineral product manufacturing	9648.90	Particulate matter; benzene; formaldehyde
Transportation equipment manufacturing	42,223	205,020	Nickel; hexavalent chromium; lead	Petroleum and coal product manufacturing	5718.41	Particulate matter; benzene; nickel
Paper manufacturing	18,307	28,530	Formaldehyde; lead; arsenic	Wood product manufacturing	5571.53	Particulate matter; formaldehyde; benzene
Fabricated metal product manufacturing	10,676	25,140	Nickel; hexavalent chromium; lead	Chemical manufacturing	4416.50	Particulate matter; benzene; dichloromethane
Wood product manufacturing	4102	9770	Formaldehyde; arsenic; benzene	Food manufacturing	3112.15	Particulate matter; formaldehyde
Machinery manufacturing	1523	7650	Nickel; lead	Transportation equipment manufacturing	1360.71	Particulate matter; tetrachloroethylene; trichloroethylene
Plastics and rubber product manufacturing	3891	6370	Lead; hexavalent chromium; 1,3-butadiene	Plastic and rubber product manufacturing	731.92	Particulate matter; tetrachloroethylene; trichloroethylene

Values for estimates of use were rounded to the nearest 10th. Top carcinogens used and released represent the three carcinogens reportedly used and released in the largest quantities, listed in decreasing order

Facilities in the chemical manufacturing sector ranked first among all sectors for reported carcinogen use, using more than 10 million t in the 5-year period analyzed (Table 1). The primary metal manufacturing and petroleum and coal products manufacturing sectors ranked second and third for total reported carcinogen use with approximately 4.7 million t and 2 million t, respectively (Table 1). The chemical manufacturing and primary metal manufacturing sectors together accounted for 84% of all carcinogen use across all sectors.

Facilities in the primary metal manufacturing sector reported the release of 23,166 t of carcinogens into the air, the most out of any sector (Table 1). Facilities in this sector released approximately 30% of the total carcinogenic emissions from all sectors. Other significant carcinogen emissions were reported by the paper manufacturing and mining sectors, with 12,130 and 9875 t, respectively (Table 1).

The largest number of workers was employed on average in the primary metal manufacturing sector (*n* = 132,401) and the transportation equipment manufacturing sector (*n* = 42,223) (Table 1). Some of the carcinogens reported in the greatest quantities among all industry sectors were lead, nickel, and benzene (Table 1). Particulate matter was reportedly released by facilities in every sector and in the largest amounts (Table 1).

Trends for facilities reporting estimated carcinogen use and release, by industry sector and year, are shown in Tables 2 and 3, respectively. The largest reported increase in the use of carcinogens from 2011 to 2015 occurred in the petroleum and coal product manufacturing sector, which increased by 14% (Table 2). All other industrial sectors, out of the top 10 analyzed, appeared to experience a decrease in carcinogen use during the study period (Table 2). The largest decreases in carcinogen use were reported by the transportation equipment manufacturing sector at 24%, as well as the primary metal manufacturing and plastic and rubber product manufacturing sector, each at 13% (Table 2). Overall, there was an observed decrease in the use of carcinogens across all industrial sectors by 8% (Table 2).

**Table 2:** Total estimated use and percent change, by industrial sector, ranked by use, TRA program 2011–2015

Sector	2011		2012		2013		2014		2015		Percent change
	Number of facilities	Estimated use	Number of facilities	Estimated use	Number of facilities	Estimated use	Number of facilities	Estimated use	Number of facilities	Estimated use	
Chemical manufacturing	47	2,624,120	56	1,630,370	52	2,554,800	53	2,075,200	50	1,584,060	− 6
Primary metal manufacturing	54	1,741,390	57	752,800	53	751,350	49	749,690	51	754,400	− 13
Petroleum and coal product manufacturing	6	297,750	6	401,850	6	357,850	6	457,290	6	462,740	14
Mining (except oil and gas)	17	129,800	18	132,660	18	137,890	17	136,020	16	121,940	− 1
Transportation equipment manufacturing	52	177,590	47	9690	48	4870	54	6110	54	6760	− 24
Paper manufacturing	14	5710	15	5700	15	5710	14	5710	12	5700	< − 1
Fabricated metal product manufacturing	69	5990	61	5320	63	5060	61	4450	55	4330	− 7
Wood product manufacturing	11	2520	9	2530	11	1650	8	1650	10	1420	− 11
Machinery manufacturing	6	1230	6	2310	7	1510	7	1340	6	1260	− 5
Plastics and rubber product manufacturing	20	1750	20	1870	18	1130	15	530	17	1100	− 13
Other	58	2780	49	3420	50	2100	48	1650	49	1690	− 11
Total (all)	354	4,990,630	344	2,948,520	341	3,823,920	332	3,439,640	326	2,945,400	− 8

Values for estimates of use were rounded to the nearest 5th. Percent change was calculated by dividing the slope of the trend line generated (for each sector’s use by year) by its intercept and multiplying by 100%

**Table 3:** Total releases and percent change, by industrial sector, ranked by releases, NPRI 2011–2015

Sector	2011		2012		2013		2014		2015		Percent change
	Number of facilities	Releases	Number of facilities	Releases	Number of facilities	Releases	Number of facilities	Releases	Number of facilities	Releases	
Primary metal manufacturing	70	4159.00	54	5109.97	66	4217.76	63	4628.18	63	5050.84	3
Paper manufacturing	19	1968.17	16	1421.79	17	2918.45	16	2937.59	15	2883.99	24
Mining (except oil and gas)	115	1944.85	97	1979.83	98	2172.16	95	2034.64	99	1743.32	− 2
Non-metallic mineral product manufacturing	106	2078.67	57	1931.92	72	1869.86	71	1891.24	68	1877.21	− 2
Petroleum and coal product manufacturing	27	1328.34	18	1118.28	16	1263.40	15	928.16	17	1080.23	− 5
Wood product manufacturing	25	1233.26	18	1054.24	22	1038.15	22	1002.29	24	1243.59	< − 1
Chemical manufacturing	75	877.04	52	817.66	69	1066.92	68	928.40	61	726.48	− 2
Food manufacturing	80	562.01	59	639.89	76	674.81	71	611.91	68	623.53	2
Transportation equipment manufacturing	76	371.08	50	233.53	74	293.94	69	210.77	71	251.39	− 8
Plastic and rubber product manufacturing	34	160.23	14	172.51	22	130.36	18	132.79	19	136.03	− 6
Other	106	217.35	59	167.16	106	181.80	108	147.77	98	118.10	− 9
Total (all)	733	14,900.00	494	14,646.78	638	15,827.61	616	15,453.74	603	15,734.71	2

Percent change was calculated by dividing the slope of the trend line generated (for each sector’s releases by year) by its intercept and multiplying by 100%

The paper manufacturing sector reported the largest increase in the release of carcinogens from 2011 to 2015, increasing by 24% (Table 3). Smaller increases in releases of less than 5% were observed for the primary metal and food manufacturing sectors (Table 3). For all other industrial sectors, out of the top 10 analyzed, an overall decrease in carcinogen release was observed (Table 3). The largest decrease in carcinogen release was reported by the transportation equipment manufacturing sector by 8% (Table 3). Overall, there was an observed increase in carcinogen emissions across all industrial sectors by about 2% (Table 3). The releases from the paper manufacturing sector appeared to drive the overall upward trend observed.

**Fig. 1:**
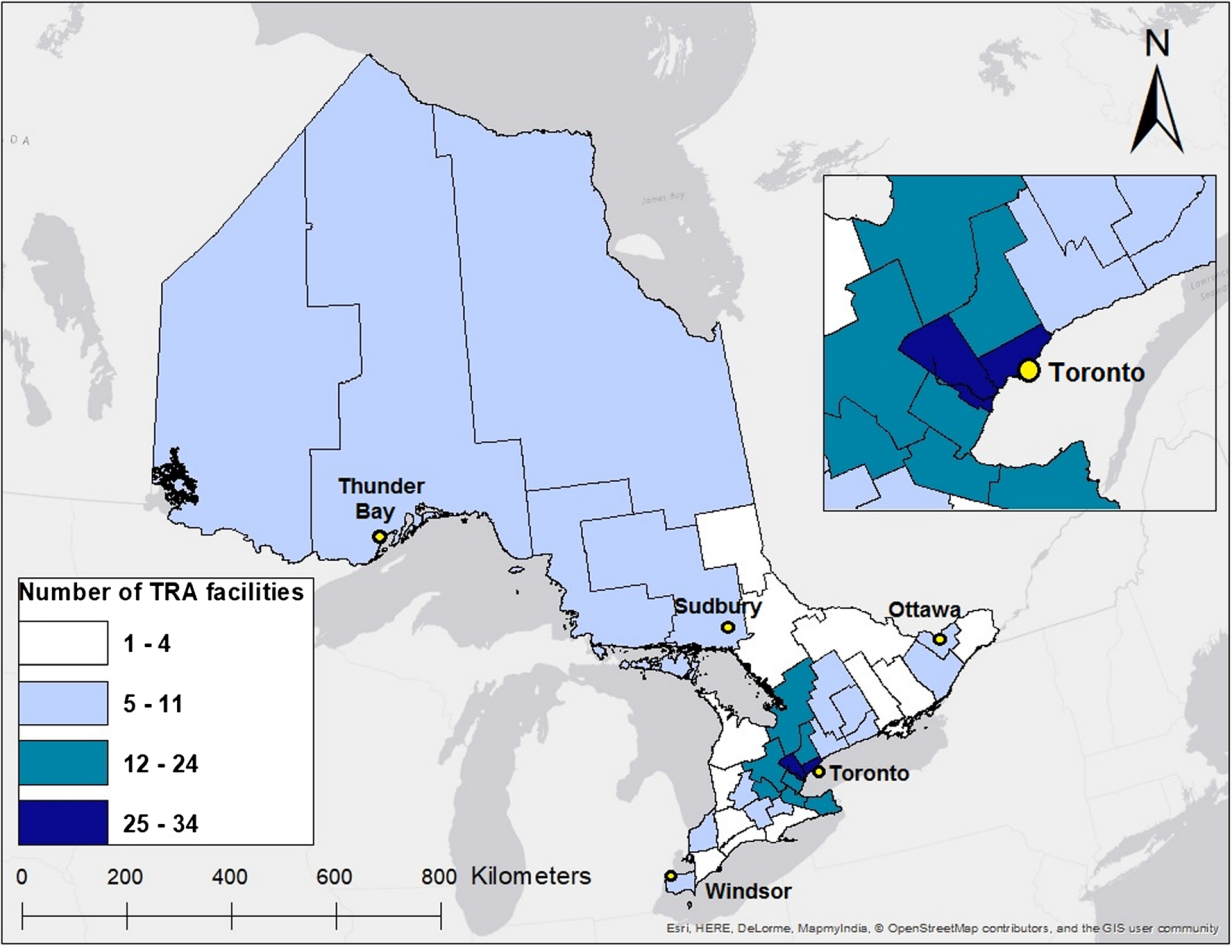
The number of industrial facilities reporting the use of carcinogens in Ontario, by Public Health Unit. Toxics Reductions Act, 2015

**Fig. 2:**
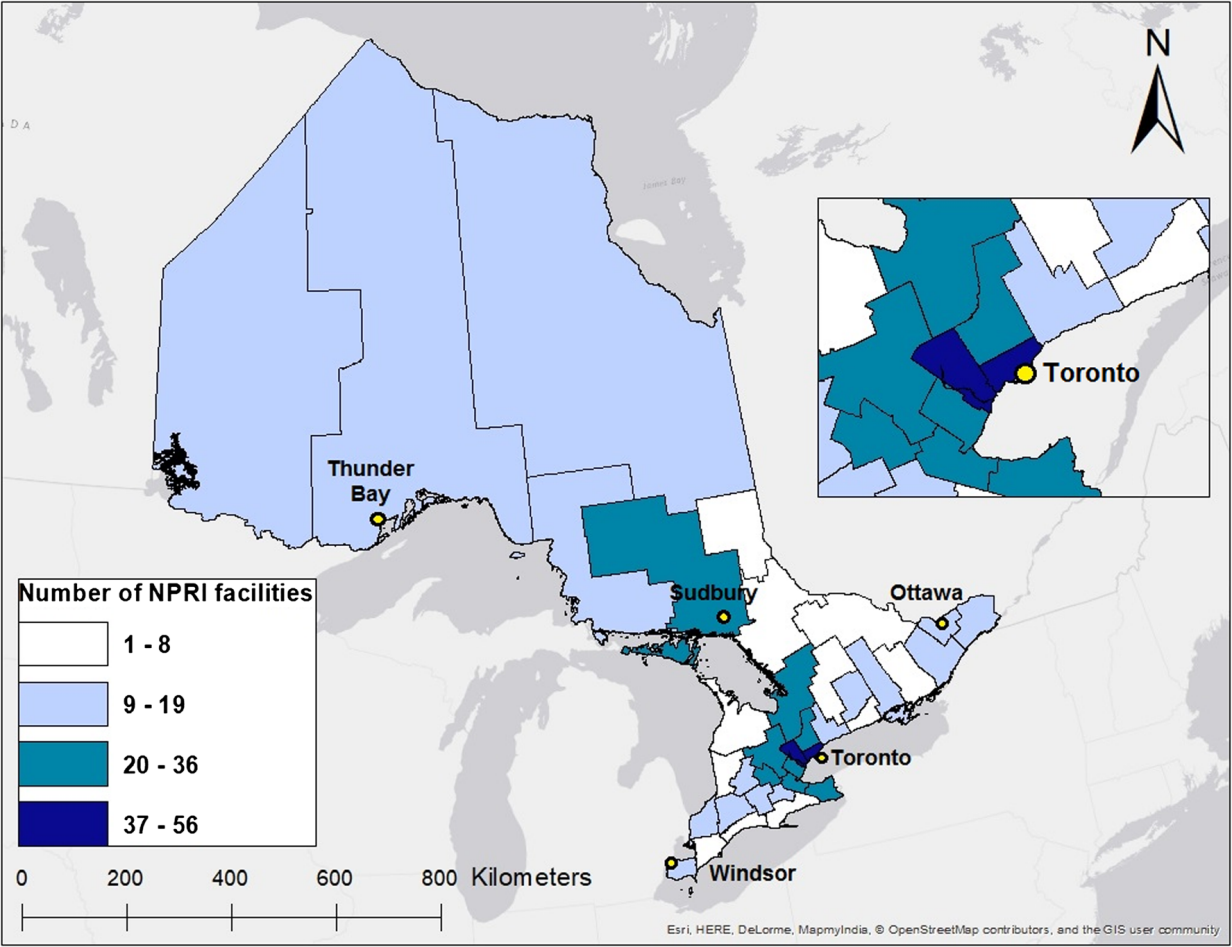
The number of industrial facilities reporting the emission of carcinogens in Ontario, by Public Health Unit. National Pollutant Release Inventory, 2015

Of the 326 industrial facilities reporting the use of carcinogens in 2015, the City of Toronto PHU and Peel Region PHU contained the largest number of industrial facilities that reported the use of carcinogens, with each containing 34 facilities (Fig. 1). Six hundred and three industrial facilities reported the release of carcinogens in 2015, with the largest number (*n* = 56) located in the City of Toronto PHU, followed by Peel Region PHU (*n* = 51) (Fig. 2). However, carcinogen use and release in 2015 by volume were highest in Lambton Health Unit (*n* = 1,343,790 t) and Sudbury and District Health Unit (*n* = 2650 t), respectively (data not shown). The chemical manufacturing and petroleum and coal product manufacturing sectors are prominent industries in Lambton Health Unit and were likely responsible for the carcinogen use observed in that PHU. In Sudbury, nickel mining remains an important industry contributing to the large quantity of carcinogenic emissions.

## Discussion

Exposures to industrial carcinogens can occur both in the workplace and in communities in the proximity of industrial facilities (Hohenadel et al. 2011). This study identified some key industry sectors in Ontario, which have also been identified in similar studies using emissions data as well as in epidemiological studies. For example, studies from the US utilizing data from the Toxics Release Inventory Program have also identified the chemical manufacturing sector as a key industry for its large release of toxic substances (Zhou and Schoenung 2009; US Environmental Protection Agency 2017). The findings by Olewiler and Dawson from Canada indicate that current trends in carcinogen releases in Ontario are similar to nationwide trends in toxic releases from over 20 years ago (Olewiler and Dawson 1998), with the primary metal manufacturing, petroleum and coal product manufacturing sectors, and mining sectors still making up the top 5 industrial sectors for releases. In a study from the UK, the manufacturing and mining sectors have been previously identified as having a large burden of occupational cancers compared to others (Hutchings et al. 2012).

We observed some notable differences between the results for carcinogen use and release, where most sectors analyzed in this study reported large quantities of carcinogens used and comparatively smaller quantities of carcinogens released. Substance use and release for any given sector may not be correlated. Substances that enter an industrial facility do not result in the direct release of those same substances by that facility as some of the substance will be contained in the facility’s product, be stored on-site, or leave the facility as wastes to landfills or releases to water, etc. Supplementing the dataset of air releases with data from facilities’ storage on-site or off-site, recycling practices, releases to water and other media could account for some of these differences and allow for a more detailed picture of the life cycle of carcinogen use from entering a facility to emission.

Other potential discrepancies in the data were due to changes in the number of facilities reporting to the TRA and NPRI. Facilities that do not meet the reporting thresholds for a given substance no longer need to report, which could reflect some of the annual changes in carcinogen use and release. To accommodate these changes, we used a slope to estimate trends in use and emission rather than calculating the differences between the first and last reporting years covered by this study.

Certain sectors appeared to report more substantial increases in releases, such as the paper manufacturing sector. Some substances may have driven the use and emission trends observed. For example, releases in particulate matter appeared to drive the trends in emissions for many sectors, whereas benzene appeared to drive trends in use (data not shown). A more detailed analysis of use and emission trends by carcinogen were previously discussed (Slavik et al. 2017.). These results illustrate the benefit of taking a sector-by-sector approach in gaining a more nuanced understanding of industrial use and emission trends in Ontario.

In addition to analyzing carcinogen use and emission by industry sector, this study examined the distribution of industrial facilities by PHU to explore potential geographic patterns in industrial sites. The largest number of industrial facilities reporting the use and release of carcinogens were observed in the city of Toronto, Ontario’s most populous city. This finding is consistent with previous research that identified significant sources of industrial pollution in Toronto, where neighbourhoods with higher emissions of toxic contaminants were also characterized by populations who were racialized or socio-economically disadvantaged (Kershaw et al. 2013). In regions where industrial facilities from specific sectors tend to be concentrated as sector hotspots, certain carcinogens that are commonly used and released in those industries are likely to be present. In Sudbury, Porcupine and other health units in northern Ontario where mining industries are concentrated, the industrial use of nickel and other carcinogenic metals is likely. Such regions could benefit from exposure reduction strategies targeted towards particular industries and carcinogens.

Lung carcinogens are among the most used in industrial facilities in Ontario (Slavik et al. 2017) and are responsible for at least 1395 occupational cancer diagnoses every year (Cancer Care Ontario, Occupational Cancer Research Centre 2017). Sectors such as the primary metal manufacturing and transportation equipment manufacturing sectors, which use particularly large quantities of carcinogens associated with these cancers, e.g., nickel, hexavalent chromium, and arsenic, ought to be prioritized for exposure reductions. These two sectors also employed the largest numbers of employees, indicating that workers in these sectors may be at risk to increased exposures. In addition, since particulate matter has been identified as a major air pollutant contributing to the environmental burden of cancer in Ontario (Cancer Care Ontario, Ontario Agency for Health Protection and Promotion 2016), efforts should be made to reduce these emissions from all sectors. By shifting towards greener chemistry alternatives, sectors could take steps to reduce, substitute, or eliminate the use and release of hazardous industrial pollutants by altering production processes or redesigning products and systems (Thorpe and Rossi 2007).

### Study limitations

One limitation of this study is that the amount of toxic substances used and emitted by facilities is self-reported in both the NPRI and the TRA, though some consistency between volumes of self-reported pollutant releases by industrial facilities and pollution monitoring data have previously been found (De Marchi and Hamilton 2006). Datasets from the NPRI and TRA are validated by program staff and errors can be identified and corrected before publication of the datasets. Due to the nature of self-reported data, there may be cases where reported carcinogen use or emissions do not reflect true use or releases by the facilities.

In addition, there are limitations in the reporting requirements of the TRA program for toxic substance use. The program allows for the reporting of use quantities by industrial facilities as ranges as opposed to absolute quantities, which limits the analysis to estimates. Another limitation of most environmental reporting programs is the fact that only larger industrial facilities meeting specific use and release thresholds are required to report (Dolinoy and Miranda 2004), potentially leaving out a significant source of emissions from smaller facilities. Therefore, it is likely that both the use and the emission of industrial carcinogens are actually much higher than what is indicated by such databases (Simmons 2013).

There are also limitations in applying carcinogen use and release as indicators of potential exposure since numerous other factors impact a worker’s exposure to chemicals. These other factors, including exposure controls such as safe work practices and personal protective equipment, were not examined in this study. It should also be noted that high sector emissions do not necessarily equate to high cancer risk since contaminants are dispersed and are often transported across large distances in the environment (Li and Ma 2016). There are also issues with identifying the timing of exposures and the long latency period of cancer that make it difficult to link particular cancers to particular carcinogenic exposures (Brody and Rudel 2003).

## Conclusions

Databases like the TRA and NPRI can be used for surveillance to provide estimates of industrial carcinogen use and release when detailed exposure assessments and routine environmental monitoring are not feasible. A major strength of the approach used in this study is the combination of descriptive and quantitative approaches to assess industrial trends in the province by both characterizing potential exposures in sectors of concern and quantifying changes in carcinogen use and release. The methods are also highly transferable to other jurisdictions where similar environmental reporting databases exist and concerns over toxics use and emission are present.

There remain opportunities to reduce the use and release of carcinogens in many Ontario industries. The TRA program is still in its infancy and future analyses drawing from more datasets will be able to better indicate longer-term trends in carcinogen use and release. The findings from this study may inform future efforts to quantify levels of exposures in particular industry sectors or geographic regions where those industries are present. While this study does not attempt to draw conclusions on the risk of developing cancer in workers and populations residing near certain industries, it demonstrates the need to prioritize exposure prevention strategies in particular sectors where the most carcinogens are used and emitted.

## References

[CR1] Brody JG, Rudel RA (2003). Environmental pollutants and breast cancer. Environ Health Perspect.

[CR2] Canadian Cancer Society. (2014). About half of cancers can be prevented. Available at: http://www.cancer.ca/en/about-us/for-media/media-releases/national/2014/world-cancer-day-2014/?region=on (Accessed October 1, 2017).

[CR3] Canadian Cancer Society’s Advisory Committee on Cancer Statistics. (2017). Canadian Cancer Statistics 2017. Toronto: Canadian Cancer Society. ISSN 0835-2976.

[CR4] Cancer Care Ontario, Occupational Cancer Research Centre. Burden of occupational cancer in Ontario: Major workplace carcinogens. Toronto, ON: Queen’s Printer for Ontario, 2017. ISBN 978-1-4868-0415-3.

[CR5] Cancer Care Ontario, Ontario Agency for Health Protection and Promotion. (2016). Environmental Burden of Cancer in Ontario. Toronto, ON: Queen’s Printer for Ontario. ISBN 978-1-4606-8367-5.

[CR6] Chakraboty J (2004). The geographic distribution of potential risks posed by industrial toxic emissions in the U.S.. Journal Of Environmental Science & Health, Part A: Toxic/Hazardous Substances & Environmental Engineering.

[CR7] Commonwealth of Massachusetts (2014). Reporting year 2012: toxics use reduction information release.

[CR8] De Marchi S, Hamilton JT (2006). Assessing the accuracy of self-reported data: an evaluation of the toxics release inventory. Journal of Risk and Uncertainty.

[CR9] Dolinoy DC, Miranda ML (2004). GIS modeling of air toxics releases from TRI-reporting and non-TRI-reporting facilities: impacts for environmental justice. Environ Health Perspect.

[CR10] Driscoll T, Steenland K, Nelson DI, Prüss-Üstün A, Campbell-Lendrum DH, Corvalán CF (2004). Occupational carcinogens: assessing the environmental burden of disease at national and local levels.

[CR11] Environment and Climate Change Canada. (2016). National Pollutant Release Inventory. Available at: https://www.ec.gc.ca/inrp-npri/ (Accessed 15 Sep 2017).

[CR12] Environment and Climate Change Canada. (2017). National Pollutant Release Inventory (NPRI)—Bulk Data. Available at: http://open.canada.ca/data/en/dataset/40e01423-7728-429c-ac9d-2954385ccdfb (Accessed 15 Sep 2017).

[CR13] Government of Ontario. Toxics Reduction Act, 2009, S.O. 2009, c. 19, 2017. Available at: https://www.ontario.ca/laws/statute/09t19 (Accessed 15 Sep 2017).

[CR14] Government of Ontario. (2017). Toxics Reduction Act – Reporting. Available at: https://www.ontario.ca/data/toxics-reduction (Accessed September 15, 2017).

[CR15] Hohenadel K, Marrett L, Bukvic D, Brown J, Harris SA, Demers PA (2011). Priority issues in occupational cancer research: Ontario stakeholder perspectives. Chronic Diseases and Injuries in Canada.

[CR16] Hutchings SJ, Rushton L, with the British Occupational Cancer Burden Study G (2012). Occupational cancer in Britain: industry sector results. Br J Cancer.

[CR17] International Agency for Research on Cancer. (2017). Monographs- Classifications. Available at: http://monographs.iarc.fr/ENG/Classification/ (Accessed 1 Sep 2017).

[CR18] Jacobs MM, Massey RI, Tenney H, Harriman E (2014). Reducing the use of carcinogens: the Massachusetts experience. Reviews on Environmental Health.

[CR19] Kershaw S, Rinner C, Gower S, Campbell M (2013). Identifying inequitable exposure to toxic air pollution in racialized and low-income neighbourhoods to support pollution prevention. Geospatial Health.

[CR20] Li P-C, Ma H-w (2016). Using risk elasticity to prioritize risk reduction strategies for geographical areas and industry sectors. J Hazard Mater.

[CR21] Lim S-R, Lam CW, Schoenung JM (2010). Quantity-based and toxicity-based evaluation of the US Toxics Release Inventory. Journal of Hazardous Materials.

[CR22] Mannetje A, Kromhout H (2003). The use of occupation and industry classifications in general population studies. International Journal of Epidemiology.

[CR23] Mennis J (2002). Using geographic information systems to create and analyze statistical surfaces of population and risk for environmental justice analysis. Social Science Quarterly.

[CR24] Mennis JL, Jordan L (2005). The distribution of environmental equity: exploring spatial nonstationarity in multivariate models of air toxic releases. Annals of the Association of American Geographers.

[CR25] Neumann CM, Forman DL, Rothlein JE (1998). Hazard screening of chemical releases and environmental equity analysis of populations proximate to toxic release inventory facilities in Oregon. Environmental Health Perspectives.

[CR26] Olewiler, N. D., & Dawson, K. (1998). Analysis of national pollutant release inventory data on toxic emissions by industry: technical committee on business. *Taxation*.

[CR27] Ontario Ministry of Health and Long-Term Care. (2014). Health services in your community. Available at: http://www.health.gov.on.ca/en/common/system/services/phu/ (Accessed 15 Sep 2017).

[CR28] Premji S, Bertrand F, Smargiassi A, Daniel M (2007). Socio-economic correlates of municipal-level pollution emissions on Montreal Island. Canadian Journal Of Public Health.

[CR29] Pulles T (2008). Quality of emission data: community right to know and national reporting. Environmental Sciences.

[CR30] Ringquist, E. J. (1997). Equity and the distribution of environmental risk: the case of TRI facilities. *Social Science Quarterly*, 811–829.

[CR31] Setton E, Veerman B, Erickson A, Deschenes S, Cheasley R, Keller C (2015). Identifying potential exposure reduction priorities using regional rankings based on emissions of known and suspected carcinogens to outdoor air in Canada. Environmental Health.

[CR32] Simmons G (2013). Clearing the air? Information disclosure, systems of power, and the National Pollution Release Inventory. McGill Law Journal.

[CR33] Slavik, C., Kalenge, S., & Demers, P. (2017). Recent trends in the industrial use and emission of known and suspected carcinogens in Ontario, Canada. *Reviews on Environmental Health*. 10.1515/reveh-2017-0021 28926342

[CR34] The International Agency for Research on Cancer (IARC). (2016). IARC monographs: outdoor air pollution Volume 109. Geneva: World Health Organization. Available at: http://monographs.iarc.fr/ENG/Monographs/vol109/mono109.pdf (Accessed 1 Oct 2017).

[CR35] Thorpe B, Rossi M (2007). Require safer substitutes and solutions: making the substitution principle the cornerstone of sustainable chemical policies. New Solutions: a Journal of Environmental and Occupational Health Policy.

[CR36] US Environmental Protection Agency. (2017). Comparing industry sectors in the 2015 TRI National Analysis. Available at: https://www.epa.gov/trinationalanalysis/comparing-industry-sectors-2015-tri-national-analysis (Accessed 15 Sept 2017).

[CR37] Wong S, Coates A, To T (2016). Exposure to industrial air pollutant emissions and lung function in children: Canadian Health Measures Survey, 2007 to 2011. Health Reports.

[CR38] Zhou X, Schoenung JM (2009). Combining US-based prioritization tools to improve screening level accountability for environmental impact: the case of the chemical manufacturing industry. Journal of Hazardous Materials.

